# Supplementary material for: Unraveling the power of NAP-CNB’s machine learning-enhanced tumor neoantigen prediction
Source: eLife. 2025 Mar 11;13:RP95010. doi: 10.7554/eLife.95010 (PMC11896607; doi:10.7554/eLife.95010)
Supplement: Figure 1—source data 1. [file elife-95010-fig1-data1.zip › Figure 1/1C/figure 1C .pdf]

The top rows show the immune response of vaccinated animals after stimulation with the indicated peptides, while the bottom rows display the response of non-vaccinated animals after restimulation with the same peptides

The columns show to duplicates of different animals i.e. column 1 and 2 correspond to different measurements of the same animal. Groups of 5 animals were tested for each condition

Plate 1:

a line and b line: controls non-immunized animals tested against TRP2

c line (immunized),**d** line (non-immunized) mice: tested against **WIZ**
e line (immunized),**f** line (non-immunized) mice: tested against **LRRC28**
g line (immunized),**h** line (non-immunized) mice: tested against **PNP**

A11,B11,C11,D11,E11: positive Control (TRP2 + anti-CD3CD28)

Plate 2:

a line (immunized),**b** line (non-immunized) mice: tested against **ADAR**
c line (immunized),**d** line (non-immunized) mice: tested against **MIX**
e line (immunized),**f** line (non-immunized) mice: tested against **HERC6**

H6,h7,h8,h9,h10: positive Control (TRP2 + anti-CD3CD28)
H1, h2, h3, h4, h5, negative controls (PBS)
