# Supplementary figures and images for: Unraveling the power of NAP-CNB’s machine learning-enhanced tumor neoantigen prediction
Source: eLife. 2025 Mar 11;13:RP95010. doi: 10.7554/eLife.95010 (PMC11896607; doi:10.7554/eLife.95010)

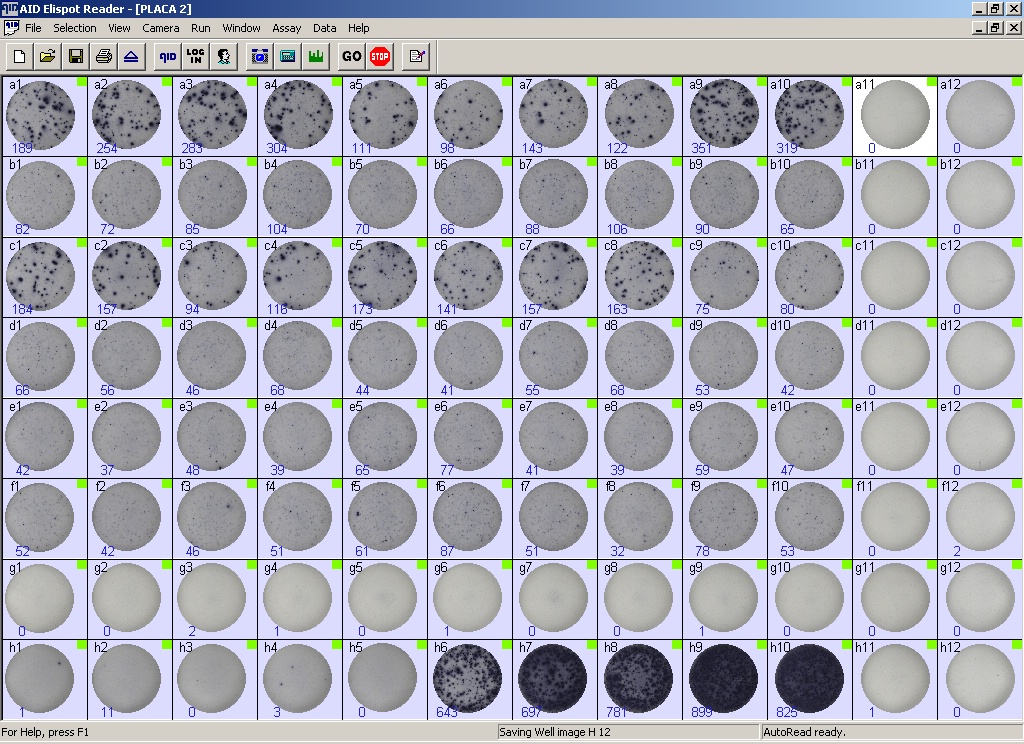

Supplement: Figure 1—source data 1. [file elife-95010-fig1-data1.zip › Figure 1/1C/1C PLATE 2.JPG]

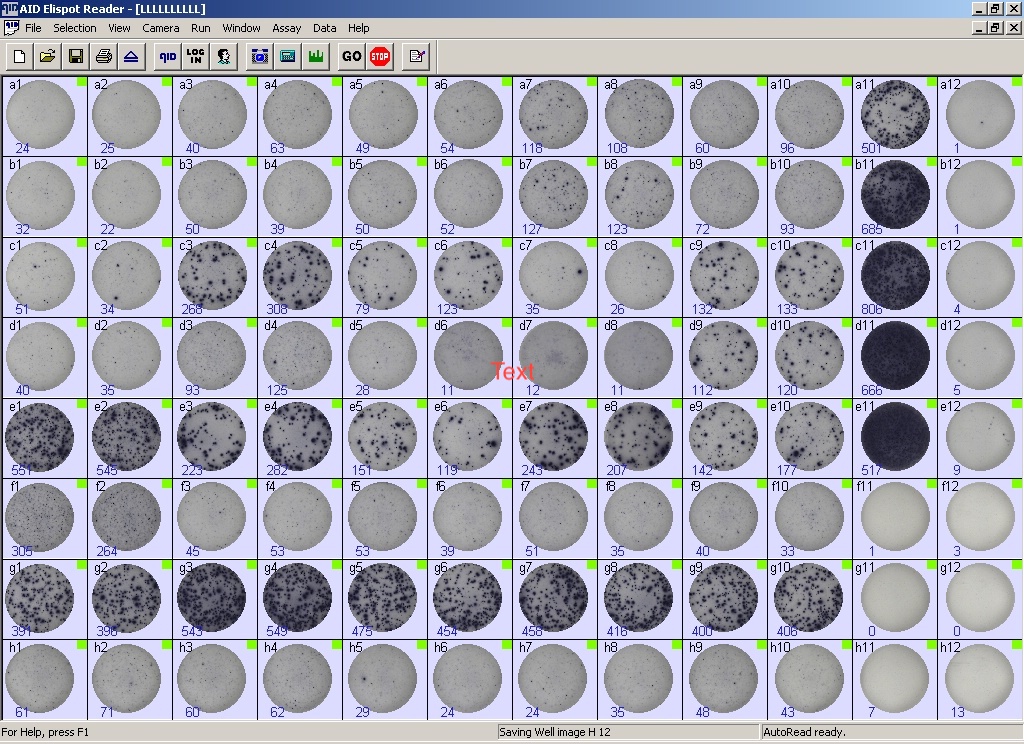

Supplement: Figure 1—source data 1. [file elife-95010-fig1-data1.zip › Figure 1/1C/1C PLATE 1.JPG]
